# Assessment of glutamatergic synaptic transmission and plasticity in brain slices: relevance to bioelectronic approaches

**DOI:** 10.1186/s42234-019-0022-2

**Published:** 2019-06-10

**Authors:** Eric H. Chang, Samantha T. Carreiro, Stephen A. Frattini, Patricio T. Huerta

**Affiliations:** 1 Laboratory of Immune & Neural Networks, Institutes of Molecular Medicine and Bioelectronic Medicine, Feinstein Institutes for Medical Research, Northwell Health, 350 Community Drive, Manhasset, NY 11030 USA; 2 Laboratory of Biomedical Science, Institute of Bioelectronic Medicine, Feinstein Institutes for Medical Research, Northwell Health, 350 Community Drive, Manhasset, NY 11030 USA; 3 Nimbus Therapeutics, 130 Prospect Street, Suite 301, Cambridge, MA 02139 USA; 4 Department of Molecular Medicine, Zucker School of Medicine at Hofstra/Northwell, 500 Hofstra Blvd, Hempstead, NY 11549 USA

**Keywords:** Uridine, Nucleoside, LTP, Synaptic plasticity, Glutamate, NMDA

## Abstract

**Background:**

Glutamatergic neurons represent the largest neuronal class in the brain and are responsible for the bulk of excitatory synaptic transmission and plasticity. Abnormalities in glutamatergic neurons are linked to several brain disorders and their modulation represents a potential opportunity for emerging bioelectronic medicine (BEM) approaches. Here, we have used a set of electrophysiological assays to identify the effect of the pyrimidine nucleoside uridine on glutamatergic systems in ex vivo brain slices. An improved understanding of glutamatergic synaptic transmission and plasticity, through this type of examination, is critical to the development of potential neuromodulation strategies.

**Methods:**

Ex vivo hippocampal slices (400 μm thick) were prepared from mouse brain. We recorded field excitatory postsynaptic potentials (fEPSP) in the CA1’s stratum radiatum by stimulation of the CA3 Schaeffer collateral/commissural axons. Uridine was applied at concentrations (3, 30, 300 μM) representing the physiological range present in brain tissue. Synaptic function was studied with input-output (I-O) functions, as well as paired-pulse facilitation (PPF). Synaptic plasticity was studied by applying tetanic stimulation to induce post-tetanic potentiation (PTP), short-term potentiation (STP) and long-term potentiation (LTP). Additionally, we determined whether uridine affected synaptic responses carried solely by n-methyl-d-aspartate receptors (NMDARs), particularly during the oxygen-glucose deprivation (OGD) paradigm.

**Results:**

The presence of uridine altered glutamatergic synaptic transmission and plasticity. We found that uridine affected STP and LTP in a concentration-dependent manner. Low-dose uridine (3 μM) had no effect, but higher doses (30 and 300 μM) impaired STP and LTP. Moreover, uridine (300 μM) decreased NMDAR-mediated synaptic responses. Conversely, uridine (at all concentrations tested) had a negligible effect on PPF and basal synaptic transmission, which is mediated primarily by α-amino-3-hydroxy-5-methyl-4-isoxazolepropionic acid receptors (AMPARs). In addition, uridine (100 μM) exerted a protective effect when the hippocampal slices were challenged with OGD, a widely used model of cerebral ischemia.

**Conclusions:**

Using a wide set of electrophysiological assays, we identify that uridine interacts with glutamatergic neurons to alter NMDAR-mediated responses, impair synaptic STP and LTP in a dose-dependent manner, and has a protective effect against OGD insult. This work outlines a strategy to identify deficits in glutamatergic mechanisms for signaling and plasticity that may be critical for targeting these same systems with BEM device-based approaches. To improve the efficacy of potential neuromodulation approaches for treating brain dysfunction, we need to improve our understanding of glutamatergic systems in the brain, including the effects of modulators such as uridine.

## Background

Bioelectronic medicine encompasses a set of technologies that harness the electrical nerve impulses of the body to treat disease. The current approaches have mainly focused on electrical stimulation of the peripheral nervous system, but there is also potential of employing the principles of synaptic function, synaptic plasticity, and brain biochemistry for the implementation of bioelectronic approaches in the CNS. Glutamate is the principal excitatory neurotransmitter in the brain. It is released from the presynaptic terminals of pyramidal neurons and it binds to glutamate receptors that are located in the postsynaptic neurons. There are three classes of ionotropic glutamate receptors, namely NMDARs, AMPARs and kainate receptors, which have a role not just in excitatory synaptic transmission but also in synaptic plasticity and higher cognitive functions. Importantly, abnormal elevations of glutamate can induce neurotoxicity, and because of this, glutamate has been implicated as a potential contributor to the pathogenesis of several neurodegenerative disorders. In this study, we aimed to investigate whether uridine is capable of altering glutamatergic synaptic transmission and synaptic plasticity with the use of ex vivo hippocampal slices and electrophysiological recordings. The hippocampal slice is an ideal preparation because it maintains many of the functions that neurons perform in vivo and it preserves the local synaptic circuitry. Therefore, brain slices are a good system in which to evaluate the molecular changes associated with drug treatment or by external neuromodulation, such as via direct current stimulation (e.g., transcranial direct current stimulation or deep brain stimulation). Moreover, hippocampal slices are able to sustain glutamatergic synaptic plasticity, which is usually tested with the paradigm of LTP. Extensive research has shown that LTP represents a form of synaptic plasticity that is input-specific, associative, and widely accepted as a synaptic model of memory formation (Bliss and Lomo, 1973; Bliss and Collingridge, 1993). In addition, it has been shown that brain slices subjected to a brief OGD injury exhibit regionally selective death of pyramidal neurons in the CA1 region, and have been used to model different brain disorders (Cho et al., 2007).

To test whether glutamatergic signaling and plasticity can be affected by non-traditional neuromodulators, we applied the nucleoside uridine on ex vivo brain slices during a broad set of electrophysiological measurements. Uridine is a building block of ribonucleic acid (RNA), which makes it an essential molecule for cell metabolism. Several decades of research have shown that uridine might have other functions in brain cells, besides being a component of nucleic acids. For instance, uridine is the only source of cytidine, which is a building block of phosphatidylcholine, one of the key phospholipids within the cell membrane (Dawson 1968; Wang et al., 2007). Some studies have shown that uridine added to neuronal cultures is capable of stimulating dendritic branching, thus increasing the number of dendrites per cell. This effect is thought to result from enhancing phosphatidylcholine synthesis, which adds new cell membrane, but also from blocking the receptors that stop dendrites from growing (Pooler et al., 2005; Silei et al., 2000). Notably, it has been shown that orally administered uridine-5-monophosphate given to aged rats supports an increased release of dopamine in the striatum (35% over control level) and dendritic outgrowth, demonstrating that, even in old animals, oral uridine intake can support neurotransmitter release and dendritic branching in vivo (Wang et al., 2005). While little is known about the effects of uridine on neurophysiology, a few studies have shown that it can work as an anticonvulsant in animal models of epilepsy (Slezia et al., 2004; Zhao et al., 2006, 2008). In regards to neurotransmitter interactions, uridine has been reported to bind competitively to gamma-aminobutyric acid (GABA) receptors (Guarneri et al., 1985) and to be released following seizures (Slezia et al., 2004), suggesting a generally inhibitory effect on synapses.

Uridine supplementation has been investigated in a number of animal models for brain disease, including epilepsy (Zhao et al., 2006; Zhao et al., 2008), Huntington’s disease (Saydoff et al., 2006), traumatic brain injury (Kabadi and Maher, 2010) Parkinson’s disease (Cansev et al., 2008), cognitive deficit (De Bruin et al., 2003), amyotrophic lateral sclerosis (Amante et al., 2010), and depression-like syndromes (Carlezon et al., 2002, 2005). Together, these results suggest that uridine is an attractive therapeutic candidate in the treatment of several brain illnesses and has an effect on brain function (Wurtman et al., 2010), although the neurophysiological basis of this effect remains to be elucidated.

Circulating plasma levels of uridine in humans range from 3 to 8 μM, but can reach concentrations of 150 μM under multiple dosing regimens (van Groeningen et al., 1991; Weinberg et al., 2010). Basal plasma uridine levels in rodents are comparable to those in humans, but within the brain, concentrations can reach the 100–300 μM range, with maximal concentrations > 350 μM after intraperitoneal dosing (Amante et al., 2010). Based on these prior findings, we decided to test three different concentrations (3 μM, 30 μM, 300 μM) of uridine for their ability to alter glutamatergic transmission and plasticity. We find that basal synaptic transmission is unaltered by the three tested concentrations, but long-term synaptic plasticity is impaired at the two higher concentrations (30 μM and 300 μM). Through the pharmacological isolation of NMDAR-mediated responses, we identify that uridine has specific effects on NMDARs in the hippocampus. We also find that uridine (100 μM) has a protective effect in an ex vivo model of ischemia.

## Methods

### Experimental animals

All animals used in this study were female BALB/cJ mice (The Jackson Laboratory, Bar Harbor, ME) of 3–8 months of age. Mice had ad libitum access to food and water, and were maintained in strict accordance with the recommendations in the Guide for the Care and Use of Laboratory Animals of the National Institutes of Health. The local Institutional Animal Care and Use Committee (Feinstein Institute for Medical Research) approved the animal protocol. All efforts were made to minimize and ameliorate suffering and pain to animals used in this study.

#### Ex vivo hippocampal slice preparation

BALB/cJ mice were anaesthetized with isoflurane in a closed container, then immediately decapitated. The brain was quickly extracted into ice-cold (< 2 °C) artificial cerebral spinal fluid (ACSF) that contained (in mM): 126 NaCl, 26 NaHCO_3_, 10 glucose, 2.5 KCl, 2.4 CaCl_2_, 1.3 MgCl_2_, 1.2 NaH_2_PO_4_ and was continuously gassed with 95% O_2_, 5% CO_2_. Kynurenic acid (1 mM), which is a non-specific blocker of excitatory amino acid receptors, was added to the ACSF during the dissection and slicing procedures. The brain was then bisected and both hemispheres were mounted onto a block with ethyl cyanoacrylate glue. Transverse hippocampal slices (400 μm thick) were prepared using a Leica VT1200 brain slicer. Brain slices were incubated for 35 min in ACSF at 35 °C, followed by 120 min in ACSF at 24 °C. Each slice was transferred to a recording chamber, continuously perfused with ACSF at 30 °C, for electrophysiological studies.

### Hippocampal electrophysiology

Field excitatory postsynaptic potentials (fEPSP) were recorded with borosilicate glass electrodes (2–3 MΩ tip resistance) placed in CA1’s stratum radiatum at the midpoint between two bipolar stimulating electrodes (Frederick Haer & Co, Bowdoinham, ME) that were placed to activate the Schaeffer collateral/commissural axons. This setup allowed for the recording of two independent pathways (test and control) in the same slice. The initial slope of the fEPSP was used as a measure of the postsynaptic response. fEPSP responses were amplified (AM Systems 1800), digitized at 10 kHz, and stored on a PC running custom software (written with AxoBasic, Axon Instruments, Union City, CA). For obtaining I-O functions, the stimulation was reduced to a value at which no fEPSP was evoked. The stimulation was then increased incrementally to evoke steeper and larger fEPSPs. This was done until the appearance of a population spike, which reflected action potentials, generated by CA1 pyramidal cells, and defined the final point of the I-O function. The protocol for PPF involved activating the afferent axons with two stimulating pulses within a short (< 1 s) inter-pulse interval (IPI). The IPIs were (in msec): 20, 50, 100, 200, 300, and 400. The paired-pulse ratio was calculated as the slope of the second fEPSP (P2) divided by the slope of the first fEPSP (P1). For plasticity experiments, a stable baseline was obtained for at least 15 min. The baseline intensity was set to obtain a fEPSP slope that was half-maximal, as determined by I-O functions. Synaptic plasticity was induced by high-frequency stimulation (HFS), which consisted of either a tetanus train (100 Hz for 1 s) or theta burst stimulation (TBS, 10 trains of 4 pulses at 100 Hz, with 200 msec between trains). We calculated three plasticity time-points, identified as PTP (measured from 6 responses at 1 min post-HFS), STP (measured from 30 responses at 10–15 min post-HFS) and LTP (measured from 30 responses at 40–45 min post-HFS). For all LTP experiments, picrotoxin (100 μM) was added to block GABA_A_ receptors. A Good Laboratory Practice (GLP) lot of ultrapure uridine (MW = 244.2) was provided by Repligen Corporation (Waltham, MA). In order to analyze the temporal summation that occurred during the TBS, we used Origin (OriginLab, Northampton, MA) software to integrate the total depolarization area of each fEPSP response during the first TBS stimulation event.

For recording NMDAR–mediated fEPSPs, we used a magnesium-free ACSF solution containing 6-cyano-7-nitroquinoxaline-2,3-dione (CNQX, 10 μM), and glycine (30 μM). Baseline NMDAR–mediated fEPSPs were acquired and analyzed once every 20 s using WinLTP 2.01 software (WinLTP, Bristol, UK). For the OGD experiments, the brain slices were introduced into the recording chamber with ACSF + uridine (100 μM) for the indicated incubation period. At the end of the incubation period, the ACSF solution was switched to an OGD solution that was identical to the normal ACSF except that it did not contain glucose and was bubbled with 100% N_2_ instead of 95% O_2_, 5%CO_2_. This OGD solution perfused the chamber for a period of 6 min, followed by normal ACSF for the remainder of the experiment.

### Statistical analysis

Data are presented as mean ± SEM, as indicated. To examine statistical significance, which was defined as *P* < 0.05, we used factorial ANOVA, repeated measures ANOVA, and Student’s t-test in samples that were normally distributed. We also used nonparametric tests, namely Mann-Whitney U (MWU) test and Kolmogorov-Smirnov test, in samples that were not normally distributed.

## Results

### Null effect of uridine on basal synaptic transmission

**Fig. 1:**
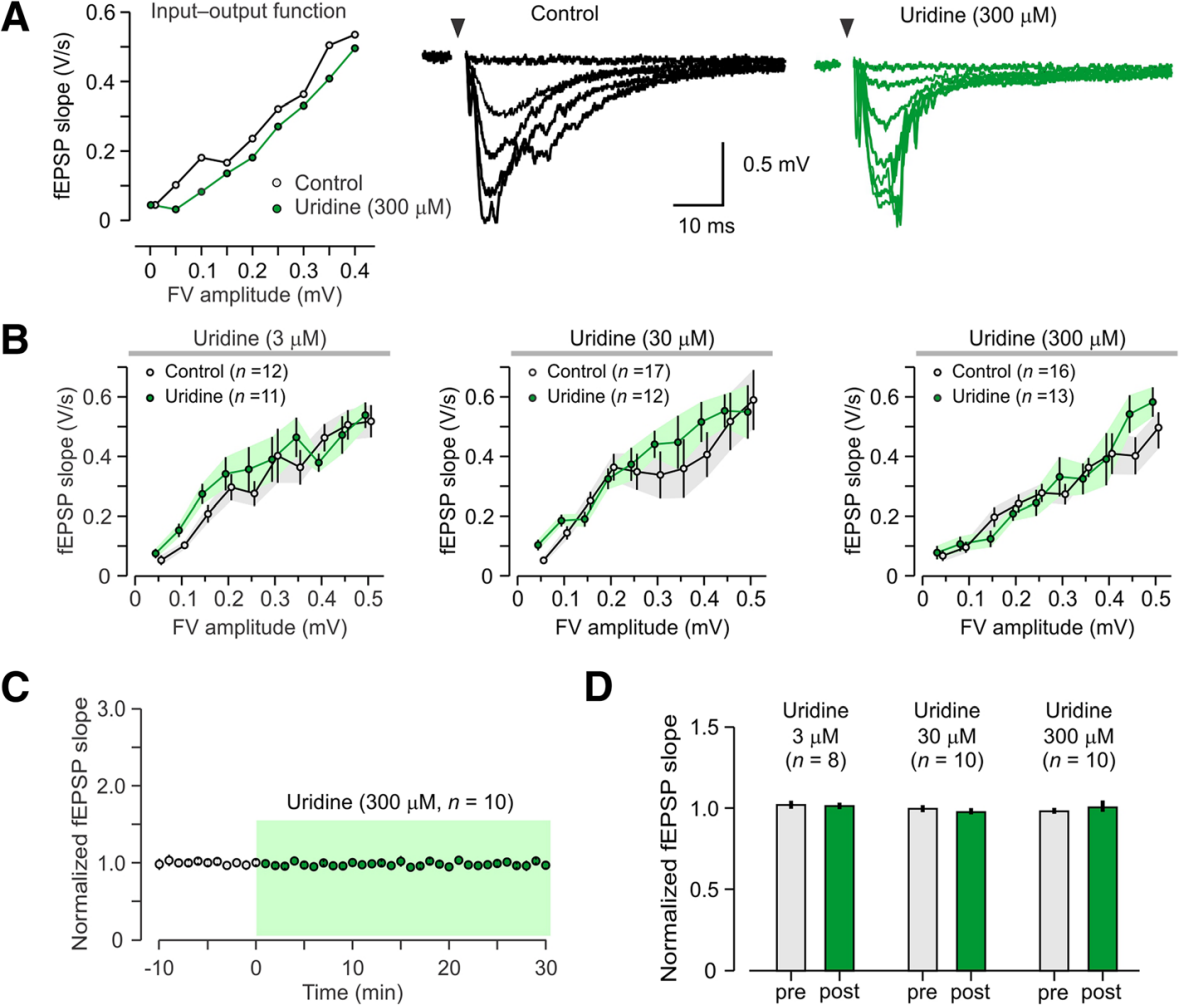
Null effect of uridine on basal synaptic transmission. **a** *Left,* representative input-output (I-O) experiments for uridine (300 μM) and control; with the amplitude of the fiber volley (FV) as the independent variable and the slope of the fEPSP as the dependent variable. *Right,* sample overlaid traces from single I-O experiments. Electrical stimulation artifacts have been removed and are marked by arrowheads. **b** Plots of I-O responses (mean ± SEM) indicate that basal synaptic transmission is not affected by any of the uridine concentrations tested. **c** Representative experiment showing that the fEPSP slope remains unchanged when uridine (300 μM) is added to the brain slice placed in the recording chamber. **d** Normalized fEPSP slope (mean ± SEM) showing that uridine (3 μM, 30 μM, and 300 μM) does not cause changes in field synaptic potentials, when measured 30 min post-application

I-O functions indicated that uridine did not have an effect on basal synaptic transmission at any of the concentrations tested (Fig. 1a, b). The range of uridine concentrations (3 μM, 30 μM, 300 μM) was chosen to represent the wide physiological range that brain tissue is exposed to in vivo, based on previous work (Amante et al., 2010). The I-O functions were compared using ANOVA with fiber volley amplitude as the repeated measure. This test showed that fEPSP slopes were similar across the range of concentrations tested: 3 μM, *F*_9, 129_ = 0.47, *P* = 0.51; 30 μM, *F*_9, 162_ = 0.46, *P* = 0.51; 300 μM, *F*_9, 162_ = 1.53, *P* = 0.24. Uridine also had no significant effect on the slope of baseline fEPSPs when introduced into the recording solution (Fig. 1c, d). These results indicate that uridine did not affect the strength of basal synaptic transmission across the population of hippocampal synapses.

### Null effect of uridine on PPF

**Fig. 2:**
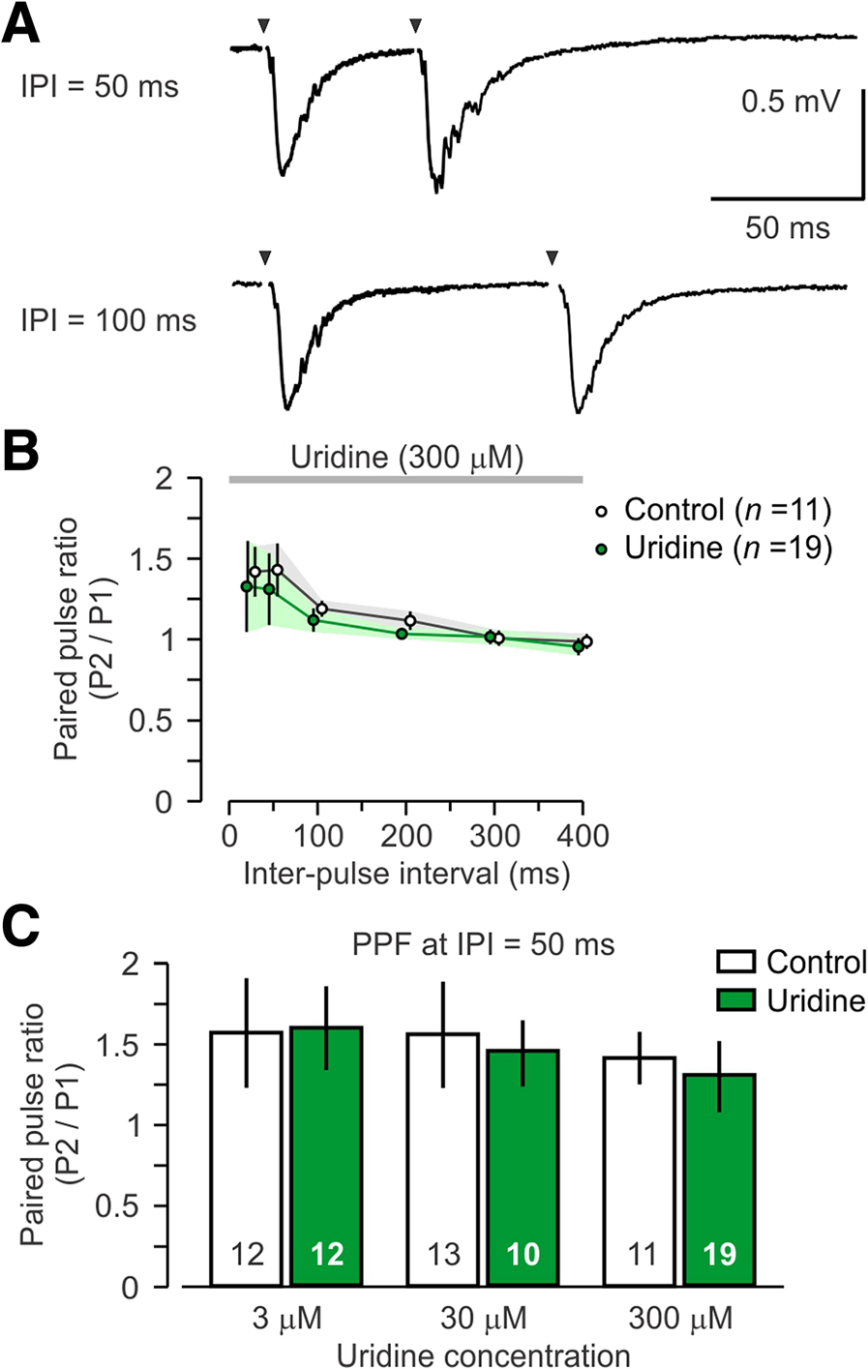
Null effect of uridine on short-term synaptic plasticity. **a** Representative traces showing paired pulse stimulation at inter-pulse intervals (IPI) of 50 ms and 100 ms from brain slices treated with high uridine (300 μM). Stimulation artifacts have been removed and are marked by arrowheads. **b** Graph showing the paired-pulse ratios (mean ± SEM) across a range of IPIs in brain slices treated with high uridine (300 μM). Ratios above 1.0 indicate paired-pulse facilitation (PPF), which is similar in the uridine and control groups; P1, slope of fEPSP in response to first pulse; P2, slope of fEPSP in response to second pulse. **c** Graphs showing the paired-pulse ratios (mean ± SEM) at a single IPI (50 ms) for uridine at three concentrations and control groups. All groups display comparable paired-pulse facilitation. Numbers within bars indicate number of brain slices per group

We tested short-term synaptic plasticity with the PPF protocol (Fig. 2a). This paradigm is designed to identify changes in the population of presynaptic terminals by using a pair of stimulating pulses within a short inter-pulse interval (Zucker 1989). PPF profiles were compared using ANOVA with inter-pulse interval as the repeated measure (Fig. 2b). This analysis showed that there were no differences in PPF across the range of concentrations tested (Fig. 2c): 3 μM, *F*_5, 115_ = 0.65, *P* = 0.80; 30 μM, *F*_5, 56_ = 3.09, *P* = 0.13; 300 μM, *F*_5, 85_ = 0.39, *P* = 0.55. This indicated that short-term synaptic plasticity was unaffected by uridine.

### Effect of uridine on STP and LTP

**Fig. 3:**
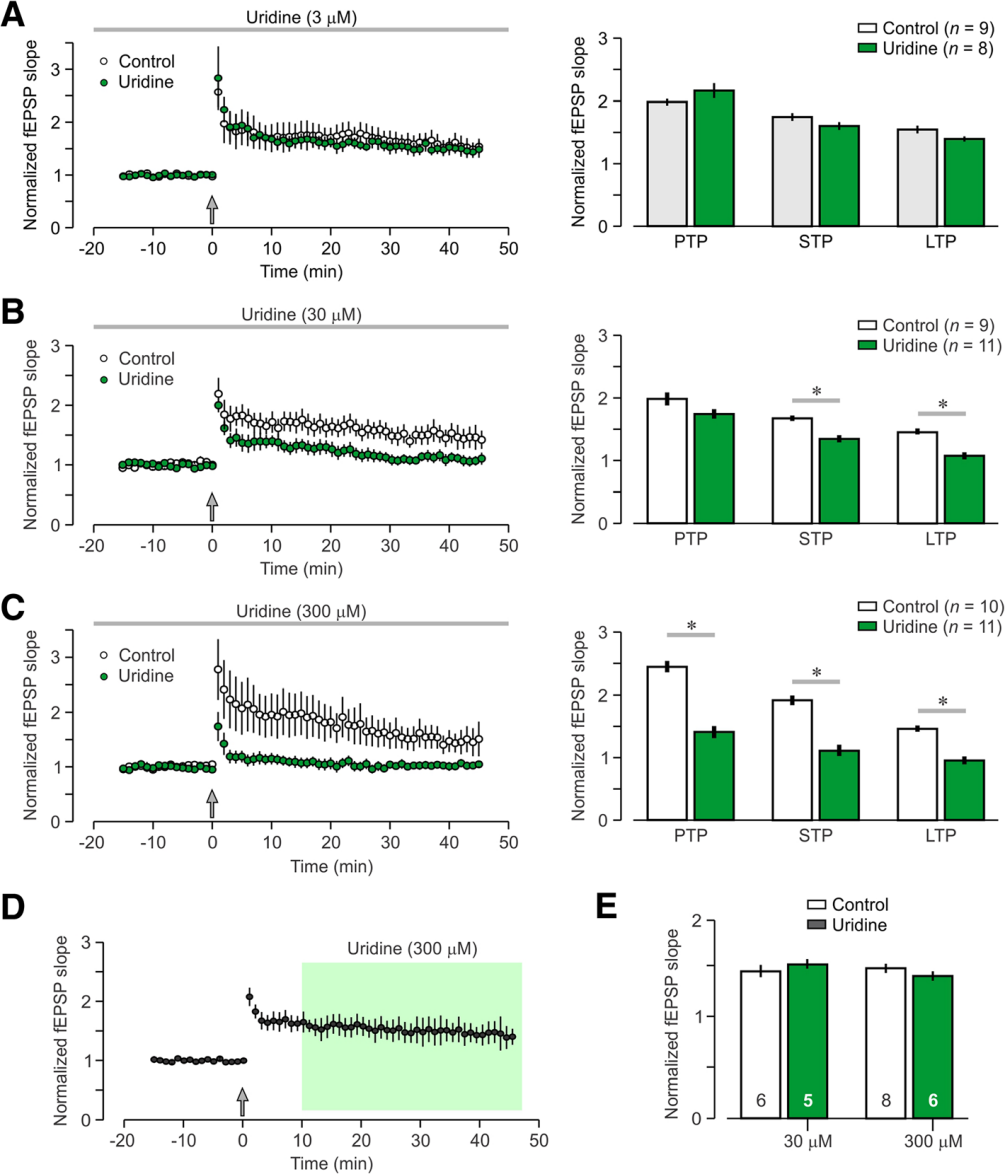
Concentration-dependent effect of uridine on the induction of long-term potentiation. Brain slices are treated with uridine and fEPSPs are recorded for at least 15 min (baseline period). Then, HFS is delivered and fEPSPs are collected for an additional 45 min. Post-tetanic potentiation (PTP) is measured 1 min post-HFS, short-term potentiation (STP) is calculated 10–15 min post-HFS, and long-term potentiation (LTP) is measured 40–45 min post-HFS. **a** *Left*, graph showing the normalized fEPSP slopes (mean ± SEM) for the uridine (3 μM) and control groups; the arrow marks HFS. *Right*, bar graphs show that uridine (3 μM) does not significantly affect any plasticity time point. **b** Uridine (30 μM) has a lowering effect on STP and LTP, but PTP is unchanged; * *P* < 0.05 (t-test). **c** Uridine (300 μM) significantly decreases PTP, STP, and LTP; * *P* < 0.05 (t-test). **d** Uridine (300 μM) has a null effect on LTP expression, when introduced 10 min post-HFS, following the induction of LTP. **e** Graphs showing the negligible effect of uridine (30 μM, 300 μM) on LTP expression. Numbers within bars indicate number of brain slices per group

Synaptic plasticity was assessed by recording fEPSPs for a baseline period (15 min) and then applying HFS, which is well-known trigger for LTP. Brain slices exposed to low uridine (3 μM) did not show significant differences in their LTP level from control brain slices (Fig. 3a, control, 155% ± 3%; uridine, 140% ± 2% of baseline values; *T* = 1.60, *P* = 0.11, t-test). There were also no differences in other plasticity time-points such as PTP (control, 198% ± 5%; uridine, 216% ± 12%; *T* = 1.96, *P* = 0.07, *t*-test) and STP (control, 175% ± 9%; uridine, 160% ± 4%; *T* = 1.34, *P* = 0.18, t-test).

Brain slices exposed to the middle level of uridine (30 μM) exhibited a significant difference in LTP (Fig. 3b, control, 144% ± 6%; uridine, 106% ± 3%; *T* = 4.30, *P* < 0.0001, t-test) and STP (control, 167% ± 6%; uridine, 134% ± 4%; *T* = 4.34, *P* < 0.0005, t-test), but no difference in PTP (control, 197% ± 9%; uridine, 174% ± 6%; *T* = 0.98, *P* = 0.33, t-test). Brain slices exposed to high uridine (300 μM) showed the most dramatic impairment in synaptic plasticity with differences in LTP (Fig. 3c, control, 147% ± 2%; uridine, 97% ± 1%; *T* = 6.55, *P* < 0.0001, t-test), STP (control, 194% ± 2%; uridine, 112% ± 1%; *T* = 5.79, *P* < 0.0001, t-test), and PTP (control, 246% ± 18%; uridine, 142% ± 7%; *T* = 5.25, *P* < 0.0001, t-test).

We next addressed the question of whether uridine affected the expression of LTP. We tested this by introducing uridine, starting at 10 min post-HFS, and measuring whether a 35-min period of drug application altered the level of potentiation (Fig. 3d). We found that uridine applied following the HFS did not have any effect on LTP expression at any of the concentrations we tested (Fig. 3e). Statistical comparison against control brain slices revealed no significant differences among groups (3 μM, *T* = 0.50, *P* = 0.62; 30 μM, *T* = 1.81, *P* = 0.09; 300 μM, *T* = 1.86, *P* = 0.07, t-tests).

### Burst analysis reveals lower total depolarization in the presence of uridine

**Fig. 4:**
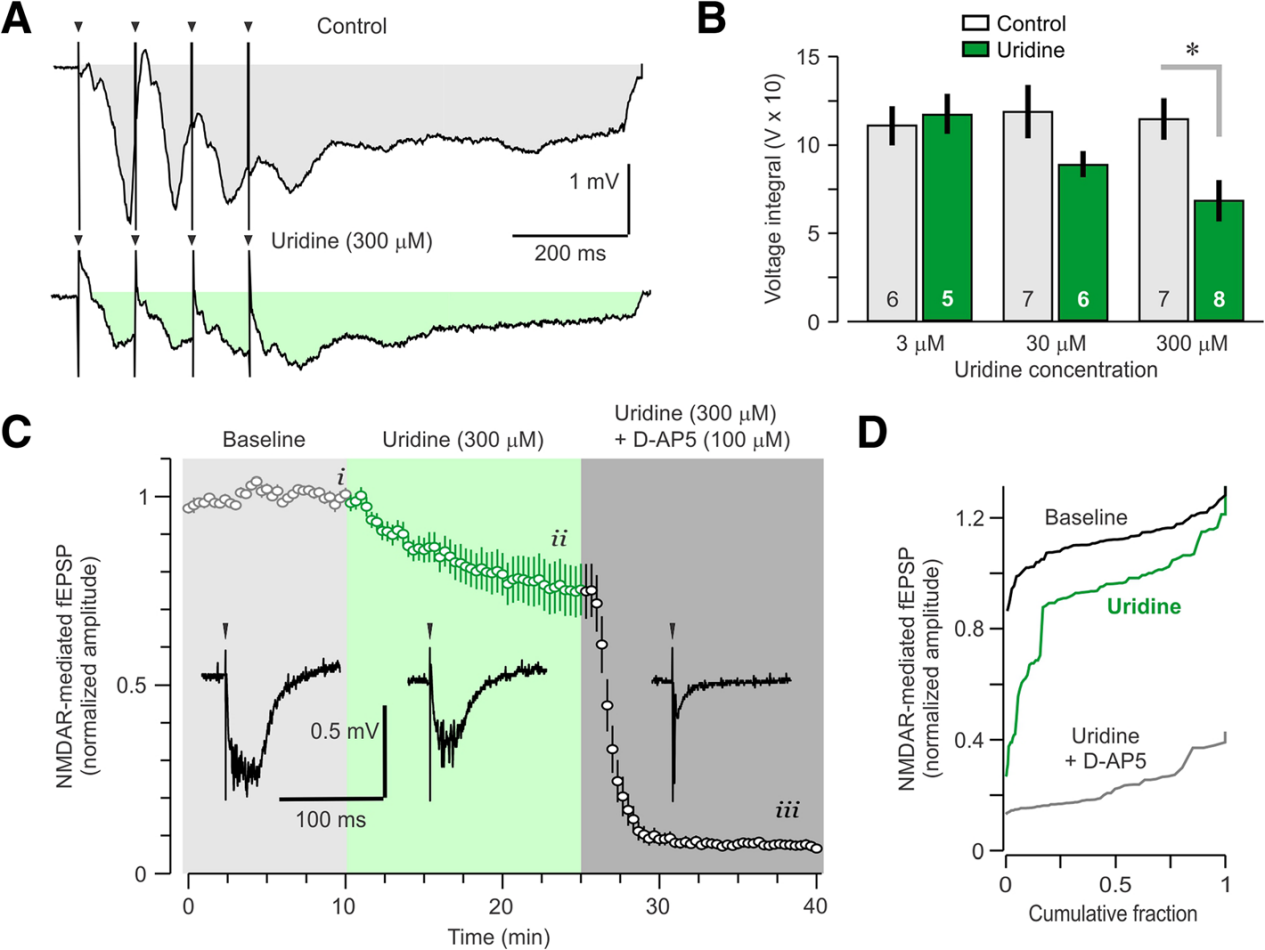
Uridine decreases NMDAR-mediated synaptic responses. **a** Representative single theta-burst stimulation (TBS) event during LTP induction. Shaded areas indicate the total depolarization measured. **b** Mean total depolarization during TBS tetanus, which is highly mediated by NMDARs, is significantly lower in the presence of uridine (300 μM); * *P* < 0.05 (MWU test). Numbers within bars indicate number of brain slices per group. **c** Plot shows mean fEPSP amplitudes of pharmacologically isolated NMDAR-mediated fEPSPs in several conditions: (***i***) baseline, no uridine (***ii***) uridine (300 μM), and (***iii***) uridine (300 μM) + D-AP5 (100 μM). Accompanying sample traces from each condition are shown in the insets. **d** Cumulative probability plots show that uridine (300 μM) significantly decrease NMDAR–mediated fEPSPs amplitudes, which are essentially eliminated in the presence of the NMDAR antagonist D-AP5

NMDAR–mediated synaptic responses have a long duration (> 100 ms) so that they summate effectively under high frequency stimulation paradigms (higher than 10 Hz). By measuring the total depolarization value during the LTP-inducing tetanic stimulation, we obtained an indirect measure of this NMDAR-mediated response. Analysis of responses during the first TBS event of each tetanus (Fig. 4a) indicated that the mean total depolarization was not different at the low concentration of uridine (3 μM; control, 110,878 ± 10,838; uridine, 117,452 ± 11,146 V, *U* = 15, *P* = 0.5, MWU test) and was reduced, but not significantly, at the middle level of uridine (30 μM; control, 118,617 ± 14,944; uridine, 88,924 ± 7398 V, *U* = 33.5, *P* = 0.079, MWU test). Interestingly, the total depolarization at the high level of uridine (300 μM) was significantly lower compared to controls (Fig. 4b; control, 114,507 ± 11,758; uridine, 68,249 ± 11,636 V, *U* = 49, *P* < 0.05, MWU test). This suggests that the high level of uridine (300 μM) impaired LTP induction, possibly by interacting with NMDARs during these high-frequency stimulation events.

### NMDAR-mediated fEPSPs are reduced in amplitude by uridine

In order to measure a potential effect of uridine on NMDARs, we recorded pharmacologically isolated NMDAR–mediated fEPSPs in the absence and presence of uridine (300 μM). Compared to typical fEPSPs, NMDAR–mediated fEPSPs were longer in duration, lower in amplitude, and were fully blocked by NMDAR antagonists (Faust et al., 2010; Izumi et al., 2006). Notably, we found that uridine (300 μM) had an inhibitory effect on the amplitude of NMDAR–mediated fEPSPs (Fig. 4c, d). Mean NMDAR-mediated fEPSP amplitudes were lowered by ~ 17% in the presence of uridine (300 μM), compared to the baseline amplitudes (baseline, 0.176 ± 0.012; uridine, 0.146 ± 0.013 mV, *D* = 0.7, *P* < 0.0001, Kolmogorov-Smirnov test). In order to verify that these fEPSPs were indeed NMDAR–mediated, we introduced the NMDAR–specific antagonist D-2-amino-5-phosphonopentanoate (D-AP5), which eliminated the fEPSP almost entirely (mean amplitude in D-AP5 = 0.0135 ± 0.003 mV). These results strongly suggest that uridine interacts with the NMDAR, acting as a partial antagonist or inhibiting agent. They also provide a mechanism to understand the LTP impairments we observed at the middle (30 μM) and high (300 μM) uridine levels.

### Protective effect of uridine against OGD

**Fig. 5:**
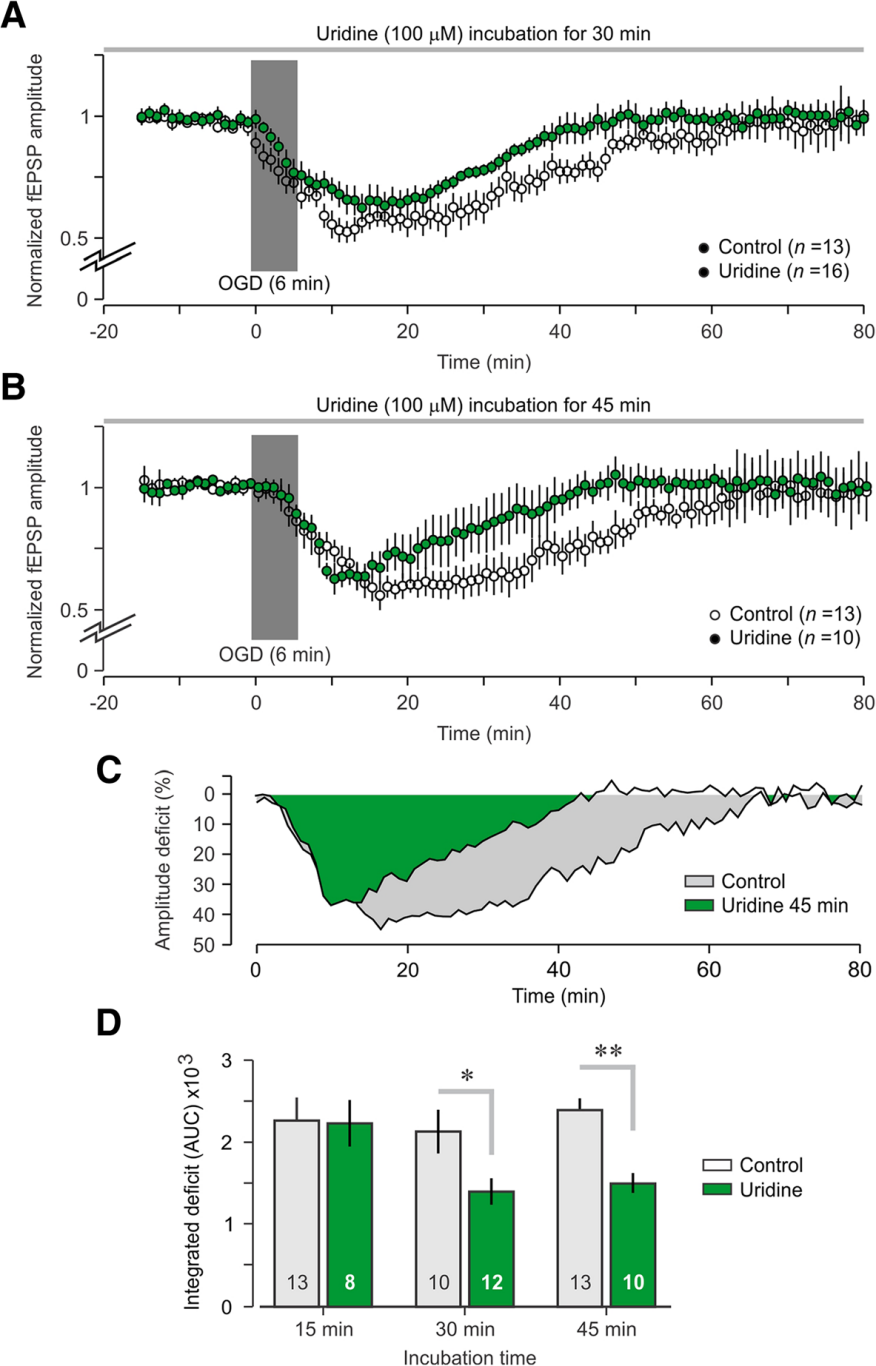
Protective effect of uridine against oxygen-glucose deprivation**. a** Graph showing normalized fEPSP amplitudes (mean ± SEM) for brain slices that are treated in uridine (100 μM) for 30 min before receiving an oxygen-glucose deprivation (OGD) insult (6 min). **b** In this set, brain slices are in uridine (100 μM) for 45 min before receiving an OGD insult (6 min). **c** Plot showing the percent amplitude deficit for the 45 min uridine group compared to the untreated control group. In the uridine group, the amplitude deficit disappears by 45 min post-OGD (vs. 65 min in control), highlighting the protective action of uridine. **d** Graph showing the total OGD deficit, which is calculated from the percent amplitude deficit plots by taking the area-under-curve (AUC) for three different incubation periods: 15 min, 30 min, and 45 min. The short incubation (15 min) is insufficient for a protective effect against 6 min OGD, while the longer incubation periods (30 min and 45 min) significantly reduce the magnitude of the OGD-induced deficit; *, *P* < 0.05; **, *P* < 0.01 (t-test). Numbers within bars indicate number of brain slices per group

To investigate the effect of uridine in an ex vivo model of brain insult, we used the OGD paradigm, which is known to trigger a rapid suppression of synaptic transmission. In this paradigm, synaptic responses fully recover (to 100% pre-insult) if the ischemic event is brief in duration. In order to obtain a reliable and reproducible OGD-induced deficit, we first ran a pilot study to test the effects of four different OGD durations; 4 min, 6 min, 8 min, and 12 min. As a result of this pilot work, we found that in our preparation a 6 min OGD challenge produced the most consistent fEPSP deficit with a mean maximum amplitude decrease of 46.0 ± 5.6% that recovered back to baseline levels after 55.7 ± 4.9 min (Fig. 5a). We then tested whether a relatively high dose of uridine (100 μM) could alter this OGD-induced decrease in fEPSP amplitude. We tested three different uridine incubation periods: 15 min, 30 min, and 45 min. The incubation period was the amount of time uridine was present in the recording chamber before the OGD insult. We found that the 15 min uridine incubation did not result in a significantly different area-under-the-curve (AUC) measurement when compared to controls (data not shown). However, the 30-min uridine incubation period resulted in a significantly reduced the deficit (Fig. 5a), and the 45-min uridine incubation was even more effective (Fig. 5b, *T* = 5.39, *P* < 0.001, t-test). In order to appropriately quantify the OGD deficit and to compare the effect of uridine between groups, we measured the AUC of amplitude-by-time plots to generate a total OGD-deficit measure (Fig. 5c). Using this measure, we found that 15-min uridine incubation did not significantly affect the magnitude of the OGD deficit (*T* = 0.09, *P* = 0.93, t-test). For the longer incubation periods, we found that the deficit was significantly reduced by uridine incubation for 30 min (Fig. 5d, *T* = 2.77, *P* < 0.01, t-test) and 45 min (Fig. 5d, *T* = 5.39, *P* < 0.001, t-test), suggesting that uridine exerted a protective effect for the synaptic population against the OGD insult.

## Discussion

Uridine has been investigated in a number of animal models for brain diseases (Amante et al., 2010; Cansev et al., 2008; De Bruin et al., 2003; Saydoff et al., 2006; Zhao et al., 2008), but despite this range of testing, the physiological effect of uridine on glutamatergic synaptic transmission and synaptic plasticity remains poorly understood. Using a set of electrophysiological assays in a brain slice preparation, our results demonstrate that uridine can impact glutamatergic synaptic transmission and synaptic plasticity in the mammalian brain. We have found that, at physiologically attainable concentrations within the brain (30 μM and 300 μM), uridine impairs long-term synaptic plasticity and inhibits NMDAR-mediated synaptic responses. Meanwhile, uridine does not have an effect on basal synaptic transmission or short-term synaptic plasticity. The protective action of uridine (100 μM) against OGD insult indicates that it acts in a beneficial way to strengthen the synaptic population by diminishing the overall OGD-induced deficit.

Together these results are a step towards understanding the effect of uridine in the brain and may be important when evaluating molecular targets for neuromodulation or in the treatment of brain disorders. For example, these results may be relevant in disorders involving excessive glutamate levels such as hyperalgesia (Sandkühler, 2009), depression (Mitani et al., 2006), epilepsy (Meldrum, 1994), and stroke (Lai et al., 2014). Notably, the Food and Drug Administration (FDA) has already approved bioelectronic interventions, such as vagus nerve stimulation (VNS), for two of these disorders, intractable depression and intractable epilepsy. While the mechanism by which VNS reduces seizure frequency or ameliorates depressive symptoms is not understood, modulation of glutamate levels within the brain is one possibility (Ben-Menachem et al., 1995; Walker et al., 1999). Further highlighting the importance of glutamatergic modulation in treating certain brain disorders, the NMDAR antagonist ketamine was very recently approved by the FDA to treat intractable depression for its rapidly acting anti-depressive effects (Krystal et al., 2019; Serafini et al., 2014).

The inhibition of NMDAR-mediated fEPSPs and lower total depolarization during tetanus in the presence of uridine (Fig. 4) suggests that the LTP impairment (Fig. 3) is due to a reduction in NMDAR-induced calcium influx, subsequently leading to lower levels of synaptic potentiation (Morris et al., 1986; Tsien et al., 1996). A previous study reported that uridine inhibited calcium uptake into synaptosomes and acted as an inhibitor of pre-synaptic NMDARs (Petrova and Gabrelian, 2008). Our results corroborate this reduction in calcium influx and extend the effect to an inhibition of NMDAR-mediated synaptic responses. While the molecular mechanism by which uridine decreases NMDAR-mediated fEPSPs is not completely understood, the fact that the synaptic effects are not detectable until the isolation of NMDAR-specific potentials (Fig. 4) suggests that uridine may act as a noncompetitive antagonist, only interacting with NMDARs when they are being excessively activated. We found that uridine reduces total depolarization under NMDAR-only stimulation, but has no effect when AMPARs are primarily being activated, as is the case during basal synaptic transmission (Fig. 1). One possibility is that uridine does not compete directly for the glutamate-binding site on NMDARs, but functions as a noncompetitive antagonist to inhibit the NMDAR glycine-binding site (Johnson and Ascher, 1987). In fact, compounds that inhibit the glycine-binding site of NMDARs have previously shown neuroprotective effects in brain slice models of ischemia (Newell et al., 1995; Warner et al., 1995), similar to what we have reported here with uridine (Fig. 5). Since our LTP experiments were performed in the presence of the GABA_A_ receptor inhibitor picrotoxin, we were not able to properly assess whether uridine interacted with the GABAergic system (Guarneri et al., 1985). However, we observed no empirical evidence that uridine displayed any GABA-mimetic effects, such as inhibiting basal synaptic transmission during our I-O tests (Fig. 1).

Excitotoxicity following a brain stroke is a primary mechanism of neuronal death and is associated with excessive glutamate that increases NMDAR-mediated calcium influx (Lai et al., 2014). While the molecular mechanisms of excitotoxicity remain poorly understood, excitatory glutamatergic transmission plays a central role in this pathophysiology. Electrophysiological measurements of glutamatergic brain activity, such as those used in this study, provide a reliable readout of neuronal and tissue viability that might be fundamental to the development of BEM treatments for stroke and other brain injuries (Rapp et al., 2015). Our observed protective effect of uridine against OGD-induced deficit (Fig. 5) may be attributed to the antagonism against NMDARs. As excitotoxic injury and activity are dependent on calcium influx via NMDARs, uridine may have attenuated this specific pathway for neuronal injury and thus allowed for a faster recovery following restoration of oxygen and glucose. It is also possible that the observed protective effect involved mechanisms that are independent of the decrease in NMDAR-mediated responses. These include possible bioenergetic effects and mitochondrial involvement (Geiger and Yamasaki, 1956). Since uridine is a pyrimidine nucleoside, the protective effects observed against OGD may be attributed to improving bioenergetics, such as elevating adenosine triphosphate (ATP) levels or enhancing glycolytic energy production. OGD triggers a rapid suppression of synaptic transmission that protects neurons by maintaining a minimal level of metabolism required for survival. This protective mechanism allows neurons to recover from ischemic insults of short duration, but prolonged ischemia (> 10 min) results in large increases in intracellular calcium, thus triggering cascades that lead irreversibly to cell death (Martin et al., 1994; Pugliese et al., 2003). Previous work has shown that uridine increases ATP levels following ischemic episodes in organs such as the heart (Aussedat, 1983) and it has also been shown to prolong the normal homeostasis of brain tissue when added to perfusion fluids (Geiger, 1958). Therefore it is possible that uridine may be elevating ATP levels and signaling via purinergic receptors such as P2X receptors. P2X receptors are cation channels that are gated by ATP and can be found in various brain regions, including the hippocampus (North, 2002; Rubio and Soto, 2001; Skaper et al., 2009). These receptors are permeable to calcium and have been implicated in LTP processes with the potential to act as facilitators or inhibitors of plasticity, depending on the context (Pankratov et al., 2009; Wang et al., 2004). Future studies that include exploration of the ATP signaling system and the use of specific purinergic antagonists should be undertaken to elucidate the mechanism for this protective effect.

Our findings point to a potential benefit of uridine in the treatment of neurological disorders where glutamatergic systems are implicated and in cases where ischemia may be involved, such as stroke or traumatic brain injury (Rapp et al., 2015). However, there is growing evidence that glutamatergic systems also play a role in the pathophysiology of major depressive disorders (Sanacora et al., 2008; Sattler and Rothstein, 2007; Zarate et al., 2005). In fact, uridine has already shown efficacy in prior studies of depression (Carlezon et al., 2002, 2005) and clinical trials for bipolar disorder (Repligen 2006, 2008). Furthermore, preclinical studies with other pyrimidines that are similar to uridine have shown antidepressant properties with effectiveness either as monotherapy (Jensen et al., 2008) or in conjunction with other compounds such as valproate (Yoon et al., 2009). Indeed, there is evidence that patients suffering from mood disorders have increased levels of glutamate in certain brain regions (Hashimoto et al., 2007) and the NMDAR may be particularly important in susceptibility for these disorders (Mundo et al., 2008). The idea that mood disorders are a product of glutamatergic dysfunction is further bolstered by evidence that mood-stabilizing drugs, such as valproate and lithium, exert neuroprotective effects against glutamate-induced excitotoxicity in neuronal cultures (Manji et al., 2000). Taken together, these pieces of evidence suggest that glutamatergic modulation of brain networks, whether by pharmacological means (e.g., uridine or ketamine) or by bioelectronic approaches (e.g., VNS), is efficacious for reducing symptoms of depression in a subset of patients.

Neuromodulation approaches using direct stimulation with implantable electrodes, such as deep brain stimulation (DBS), are a form of BEM that has been in clinical use for over two decades. DBS is effective for movement disorders, such as Parkinson’s disease, and has also been investigated for the treatment of major depression (Williams and Okun, 2013). While DBS for these indications targets dopaminergic systems, similar neuromodulation technologies can be used to target glutamatergic systems. For instance, early clinical trials of DBS targeted to the fornix of Alzheimer’s disease patients, were designed  to increase glutamatergic activity in medial and corticolimbic brain circuits, with the explicit goal of improving cognition (Nardone et al., 2015). While larger scale clinical trials of fornix DBS did not show clinical efficacy in Alzheimer’s disease (Leoutsakos et al., 2018), electrical neuromodulation of limbic structures such as the hippocampus remains an active area of investigation. Modulating hippocampal synaptic plasticity is often the goal of neurostimulation techniques, whether for the stabilization of memory decline in dementia or to ameliorate seizures in epilepsy. These emerging techniques require a thorough understanding of the excitatory brain networks and molecular targets that modulate them. Comprehensive electrophysiological testing of these circuits will improve our ability to intentionally alter them for therapeutic benefit.

As bioelectronic tools evolve and expand into CNS disorders involving glutamate, it will be important to understand the mechanisms of glutamatergic synaptic transmission and plasticity. It is also critically important to understand the role of potential neuromodulators, such as uridine, as targeted electronic interventions seek to replicate or improve upon traditional molecular targets. The electrophysiological assessment of glutamatergic systems, as demonstrated in this study, provides important foundational knowledge for the development of future BEM approaches aimed at treating a range of disorders involving glutamatergic signaling.

## Conclusions

- Electrophysiological tests performed on brain slices can be used to identify specific alterations in glutamatergic synaptic transmission and plasticity
- Uridine is a nucleoside that affects NMDAR-mediated glutamatergic transmission.
- Uridine impairs short-term and long-term synaptic plasticity.
- OGD-induced synaptic transmission deficits are ameliorated by uridine.
- An improved understanding of glutamatergic brain systems, including mechanisms of neuromodulation, will be important for any bioelectronic approaches targeting these systems.

## Data Availability

The datasets used and analyzed during the current study are available from the corresponding author on reasonable request.

## Abbreviations

ACSF: Artificial cerebral spinal fluid
AMPAR: α-amino-3-hydroxy-5-methyl-4-isoxazolepropionic acid receptor
ATP: Adenosine triphosphate
AUC: Area under the curve
BALB/c: Bagg albino, genotype c
BEM: Bioelectronic medicine
CA1: Cornus Ammonis 1 area of the hippocampus
CA3: Cornus Ammonis 3 area of the hippocampus
CNQX: 6-cyano-7-nitroquinoxaline-2,3-dione
CNS: central nervous system
D-AP5: D-2-amino-5-phosphonopentanoate
DHA: docosahexaenoic acid
FDA: Food and Drug Administration
fEPSP: field excitatory postsynaptic potential
FV: fiber volley
GABA: gamma-aminobutyric acid
GLP: Good Laboratory Practice
HFS: high-frequency stimulation
I-O: input-output
IPI: inter-pulse interval
LTP: long-term potentiation
NMDAR: n-methyl-d-aspartate receptor
OGD: oxygen-glucose deprivation
P2X: purinergic ATP-gated receptor 2X
PPF: paired-pulse facilitation
PTP: post-tetanic potentiation
RNA: ribonucleic acid
STP: short-term potentiation
TBS: theta-burst stimulation
VNS: vagus nerve stimulation
